# Traumatic Brain Injury-Related Attention Deficits in Children: A Controlled Treatment Trial with Lisdexamfetamine Dimesylate (Vyvanse)

**DOI:** 10.3390/brainsci11010117

**Published:** 2021-01-16

**Authors:** Michael G. Tramontana, Jonathan W. Prokop, Edwin Williamson, Tara Duffie, Hayden LaFever

**Affiliations:** Department of Psychiatry and Behavioral Sciences, Vanderbilt University Medical Center, The Village at Vanderbilt, Suite 2200, 1500 21st Avenue South, Nashville, TN 37212, USA; jonathanwprokop@gmail.com (J.W.P.); edwin.williamson@vumc.org (E.W.); taraduffie@icloud.com (T.D.); hayden.a.lafever@vumc.org (H.L.)

**Keywords:** traumatic brain injury, concussion, attention deficits, children, stimulant medication treatment

## Abstract

Attention deficits are among the most common and persistent impairments resulting from traumatic brain injury (TBI). This study was the first to examine the effects of lisdexamfetamine dimesylate (LDX, Vyvanse) in treating TBI-related attention deficits in children. It was an extension of a previous controlled trial with adults. This was a 12-week, randomized, double-blind, placebo-controlled, dose-titration, crossover trial. In addition to weekly safety monitoring, there were assessments on a broad range of neuropsychological and behavioral measures at baseline, 6-weeks, and 12-weeks. A total of 20 carefully selected children were enrolled, ranging from 10 to 16 years of age. The sample consisted of cases with mainly mild TBI (based on the known details regarding their injuries), but they had persisting attention deficits and other post-concussion symptoms lasting from 2 to 29 months by the time of enrollment. A total of 16 children completed the trial. One of the children withdrew due to a mild anxiety reaction while on LDX. There were no other adverse effects. Positive treatment results were found on both formal testing of sustained attention and in terms of parent ratings of attention, emotional status, behavioral controls, and various aspects of executive functioning. The findings also served to highlight broader insights into the nature of attention deficits and their treatment in children with TBI.

## 1. Introduction

New-onset or acquired attention deficits have been observed in both children and adults following traumatic brain injury (TBI). In a study by Levin et al. [1], increased rates of newly diagnosed attention-deficit/hyperactivity disorder (ADHD) were found in children post-TBI (ranging from 14.5% at 12 months to 18.3% at 24 months). The rates would have been higher if selective symptoms were considered rather than requiring that the full criteria for ADHD be present. Additional studies have established a significant causal link between TBI and attention deficits in children, with prevalence rates ranging from 20–46% and with persisting deficits lasting 4–10 years or more [2,3]. Important moderating variables have included severity and location of injury, age at the time of injury, IQ, and psychosocial factors.

The underlying mechanisms producing attention deficits post-TBI may be conceptualized in various ways. Injury to specific areas may be involved, as consistent with models of attention components and their mediation by different regions of the brain. Mirsky et al. [4] articulated a four-component model of attention (focus-execute, sustain, encode, shift) that has been applied widely in ADHD research. It also has been validated as applicable to children with TBI, with the underlying components affected to varying degrees depending on factors like severity of injury [5]. The particular pattern of impairment based on this model has also been found to differ between children with TBI versus idiopathic ADHD [6].

In a study using functional magnetic resonance imaging (fMRI), Kramer et al. [7] examined long-range outcomes with respect to attention processing in children who sustained moderate-severe TBI in early childhood versus a group of age-matched children with orthopedic injuries. The children with TBI were found to activate similar networks of brain regions relevant to attention, albeit to a significantly greater extent in particular frontal and parietal regions compared to the controls. This may be viewed as suggesting a pattern of persistent compensatory activation in response to injury of underlying components.

Treatment of attention processes with psychostimulant medication may also be conceptualized in various ways. In an fMRI study of adults with non-TBI-related ADHD, Bush et al. [8] found that psychostimulant medication (methylphenidate) produced increased activation in the dorsal anterior mid-cingulate cortex and dorsolateral prefrontal and parietal cortex, thereby, normalizing what ordinarily may be hypo-functioning of these regions in ADHD. Based on the Kramer et al. study noted above, it may be hypothesized that compensatory activation occurs, to some extent, naturally in TBI-related attention deficits, possibly especially in children. Stimulant medication may help to facilitate the process of compensatory activation, albeit in a more targeted or efficient fashion, such as by targeting dopamine transmission and synaptic plasticity in fronto-striatal regions [9]. Alternatively, stimulant medication for acquired attention deficits may serve to activate secondary or backup neural circuits relevant to attention regulation. Or, rather than activating focusing or inhibitory mechanisms, per se, there may be stimulant action on general alertness and arousal. This is especially relevant in TBI in that fatigue often arises due to increased effort or exertion needed in executing normal tasks.

A study by Tramontana and associates [10] examined the effects of lisdexamfetamine dimesylate (LDX, Vyvanse) in treating attention deficits in adults due to moderate to severe TBI. It was one of the most rigorous studies in this area and was the first controlled study using LDX with this population. Positive treatment effects were found involving various measures of sustained attention, working memory, response speed stability and endurance, and in aspects of executive functioning. No major problems with safety or tolerability were observed. Treatment was for only a six-week period (it was a 12-week, randomized, double-blind, placebo-controlled, dose-titration, crossover trial), but was enough to show impact on areas beyond narrowly defined aspects of attention. Conceivably, with more stable and regulated attention, individuals with TBI may be better able to derive benefit from other interventions, including therapies targeting other cognitive and behavioral areas affected.

The previous trial dealt with individuals with TBI ranging from 16 to 42 years of age. The present study was a similar trial but, instead, targeted children from 6 to 16 years old. Children in that age range comprise a major portion of the TBI population [11], with most of the injuries resulting from falls, recreational activities (including sports concussions), as well as motor vehicle accidents (MVAs) and pedestrian-MVAs. Some are victims of violence. Impairments in attention can have a major adverse effect on learning and behavioral adjustment. Arguably, left untreated, these deficits in children can have an even more life-altering effect on achievement and future success than with adults.

Thus far, there have been few studies examining the use of stimulant medication in treating TBI-related attention deficits in children [12,13,14,15,16]. One of the better controlled studies was a recent investigation by LeBlond and colleagues [16]. Positive outcomes were reported on a range of performance and behavioral measures after four weeks of treatment on methylphenidate (MPH) vs. placebo. For the most part, however, there have been methodological problems characterizing the studies done here, including limitations in sample size and the scope of outcomes examined. The findings have tended to be weak or inconsistent. Few studies were controlled trials. Nearly all focused specifically on MPH as the stimulant treatment. The limited nature of the studies here stands in contrast to the growing body of evidence noted above documenting the prevalence and persistence of attention deficits due to TBI in children.

The present study was intended to help address this. It was a controlled clinical trial examining the effects of LDX (Vyvanse) in the treatment of TBI-related attention deficits in children (ClinicalTrials.gov, # NCT02712996, registration as a randomized controlled trial). It followed a design and methodology similar to those in the study by Tramontana et al., albeit adapted to a child sample. However, unlike the adult trial, subject selection was extended to include cases with milder TBI. Even if the attention deficits in milder cases may not prove to be chronic, it was thought that helping to enhance functioning could have a beneficial effect on overall outcomes and limit secondary problems that might otherwise arise. Another revision was the shortening of the minimum required time post-injury from six to two months. This was intended to capture child subjects earlier in the recovery process. It was also aimed at reducing the attrition of potential cases who, otherwise, might be lost to follow-up or started on other treatments.

It was predicted that, as in the previous trial with adults, positive treatment effects would be found on a range of outcomes involving attention and various behavioral and cognitive areas affected by it. Safety and tolerability were carefully examined for LDX in this clinical application. In addition, as was done in the study with adults, treatment outcomes were examined in terms of potential moderating effects involving a broad range of pre-treatment subject characteristics. More generally, the aim of the study was to gain further insights into the nature of attention deficits and their treatment in children with TBI.

## 2. Methods

### 2.1. Subject Selection

The subject sample consisted of 20 children diagnosed with TBI-related attention deficits. The specific selection criteria were as follows.

#### 2.1.1. Inclusion Criteria

- Males and females ages 6 to 16
- TBI rated as mild/moderate/severe based on assorted factors (*Glasgow Coma Scale*, estimated post-traumatic amnesia, indications of intracranial injury on CT scan, etc.)
- TBI sustained 2–36 months earlier
- Considered to be neurologically stable (absence of lingering symptoms of confusion, disorientation, etc.)
- Persistent (>2 months) problems with focused or sustained attention
- Problems with attention/concentration rated as among the most prominent cognitive changes
- Accompanying features may include diminished arousal/speed/stamina and/or hyperactivity/impulsivity symptoms

#### 2.1.2. Exclusion Criteria

- Cases with primarily penetrating head trauma
- Pre-injury history of diagnosed ADHD
- Pre-injury history of other neurodevelopmental disorders including intellectual disabilities, major communication disorders, or autism spectrum disorder
- Unstable or serious psychiatric conditions, such as psychotic symptoms. (Concurrent problems with depression, anxiety, or post-traumatic stress disorder may be present, but are judged as stable and as not requiring pharmacologic treatment.)
- Treatment with psychotropic medication(s), including stimulants, within the past six weeks, but eligible thereafter for inclusion in the trial
- Lifetime history of stimulant abuse or dependence. Other (non-stimulant) substance abuse within the past six months.
- Tics or other contra-indications for psychostimulant use including cardiovascular disease, uncontrolled hypertension or hyperthyroidism, glaucoma, agitation, and use of a MAO inhibitor within the past six weeks. Pregnancy was also an exclusion for girls of childbearing age.
- Estimated IQ < 70
- Sensory and/or motor impairment(s) seriously limiting testing options
- Neurological conditions including uncontrolled epilepsy, degenerative disorders, brain tumor, or primary stroke
- Physical condition affecting decreased arousal, activity level, or stamina including uncontrolled hypothyroidism, severe or symptomatic anemia, autoimmune or metabolic disorders, untreated moderate/severe sleep apnea, etc.

#### 2.1.3. Recruitment Process

Children were recruited from hospitals and clinics at Vanderbilt University Medical Center. An initial review of medical records served to narrow the pool in terms of age, indications of TBI, and any contraindications. All study methods, including recruitment procedures, were approved by the Vanderbilt University Institutional Review Board (IRB) for Human Subjects Research (Project IRB# 151965, 11 February 2016).

#### 2.1.4. Screening Assessments/Enrollment

Parents of cases meeting the initial criteria from record review were contacted via letter informing them of the study and asking them to consider participating in further screening to determine eligibility. Parents were then contacted by phone to further inform them of the study and to ask permission to participate in a brief telephone screening (10–15 min) to get basic information concerning eligibility. A set script was followed, which focused mainly on whether there were cognitive problems involving attention/concentration and to determine if there were disqualifying conditions.

Next, potentially appropriate children and their parent(s) were invited to come in person for an in-depth determination of eligibility. Each underwent a semi-structured interview by the project neuropsychologist/principal investigator (MGT) to obtain more detailed information about the TBI, post-concussion symptoms, persisting problems with attention and related areas, presence of any co-morbid psychiatric conditions, and clarification of premorbid history. Additionally, rating forms were used in eliciting detailed information about current cognitive and behavioral status. This included behavior ratings on the Conners-3 assessing ADHD and related areas. Separate forms were completed by parent and child. Selection required a *T*-score of 65 or higher (+1.5 SD) on one or more of the following subscales: Inattention, Hyperactivity/Impulsivity, ADHD Inattentive Symptoms, and ADHD Hyperactive-Impulsive Symptoms. They also completed a Post-TBI Symptom Questionnaire, which further delved into mental functioning. It was a 40-item inventory spanning a total of eight cognitive domains (alertness/attention, orientation, perception, communication, mental control, thinking, memory/new learning, and specific skills). Each item was rated on a 0-3 scale of severity, with a score of 2 and higher considered as indicating a significant complaint. Subject selection required that, categorically, attention problems were rated as among the most troubling cognitive symptoms persisting since the TBI. Each case was also screened for the necessary minimum IQ of 70. That was estimated with the Vocabulary subtest of the Wechsler Intelligence Scale for Children-Fifth Edition (WISC-V) and required a scaled score of 4 or higher (M = 10, SD = 3).

Lastly, each candidate underwent a physical exam and review of medical and psychiatric history by a board-certified child and adolescent psychiatrist on the investigative team (EW). Enrollment was contingent on verifying the absence of any contra-indications for psychostimulant use, as noted above. Female patients of child-bearing age also had to have a negative urine pregnancy test. Parents were provided with information to help them guide their daughters on avoiding pregnancy and what actions should be taken if they were to become pregnant while in the study.

Upon meeting all eligibility requirements, each candidate completed an approved informed consent process (separate consent/assent procedures were used for parent and child). Financial incentives and reimbursement of travel expenses were offered for participation in the study.

Recruitment was continued until the enrollment goal of 20 cases was met (which took about two years). Thirty-four candidates completed a screening visit. Of the 14 who were not enrolled, eight did not meet the full selection criteria. The other six cases declined to participate for one reason or another (scheduling demands, reluctance to pursue medication treatment, etc.).

### 2.2. Study Design

As in the initial study, this was a randomized, double-blind, placebo-controlled, dose-titration, crossover trial. Following enrollment, each case was randomly assigned to one of two treatment sequences, alternating on whether stimulant treatment or placebo came first. Each phase was six weeks long, resulting in a total duration of 12 weeks. Comprehensive neurobehavioral assessments were performed at baseline, six weeks, and 12 weeks. Streamlined behavior ratings along with safety monitoring and medication/placebo dispensing were done during weekly visits.

### 2.3. Medication Trial

#### 2.3.1. Source

Medication was supplied by Shire Pharmaceuticals GmbH, which is the manufacturer of Vyvanse and the funding source for this investigator-initiated research trial (IIR-USA-000881). The Vanderbilt Investigational Drug Service (IDS) repackaged the active medication to provide placebo and drug capsules identical in size (the smallest available) and appearance that remained the same throughout the trial. The IDS performed medication blinding and distribution to the study staff for dispensing.

#### 2.3.2. Protocol

Enrolled children entered a pre-determined randomization scheme as designed by the IDS. They received LDX (Vyvanse) 20–70 mg or placebo for six weeks. At the end of six weeks (day 43 after treatment initiation), each subject was switched from the current agent to the alternative one. Based on manufacturer’s guidelines, no taper or washout period was deemed necessary either in switching from active drug to placebo or at termination of treatment.

#### 2.3.3. Titration

All subjects in the LDX treatment phase of the protocol began dosing at 20 mg po on study day 1 and continued that for week 1. (The usual starting dose is 30 mg for children 6 years of age and older with idiopathic ADHD, but a more conservative starting point was chosen given the off-label use in the present trial.) If tolerated without indication of medication sensitivity (such as mild increases in anxiety, insomnia, weight loss, etc.), the dose was increased to 30 mg for week 2. Thereafter, if tolerated, it was increased to 50 mg for week 3 and again for week 4 to a maximum dosage of 70 mg. Increments were scaled back to a rate of 10 mg weekly at any point if there was concern about possible medication sensitivity. Subjects remained at the maximum tolerated dose for the remainder of the trial unless they met safety endpoints for withdrawal (see below) or requested to exit the study. Cases with certain medical conditions (e.g., those with known or suspected renal dysfunction) were not to be advanced to a daily dose beyond 50 mg. If a subject tolerated lower dosing(s) but reported tolerability problems after a dose increase, the dose was titrated downward to the prior tolerated dose level. Dosing adjustments were allowed up to the start of week 6 if necessary. The same titration schedule and guidelines were applied during both the drug and placebo phases of the trial.

#### 2.3.4. Weekly Monitoring

Once started, all cases underwent weekly (+/−3 days) clinical monitoring, drug trial implementation, and safety and compliance assessments by the project medical staff. Safety monitoring included assessment of any self-reported or parent-reported adverse events (AEs), assessments of blood pressure, heart rate, and weight, as well as psychiatric symptom assessment. There were pre-defined safety endpoints, based on both medical and psychiatric AEs, that served as withdrawal criteria if met.

#### 2.3.5. De-Blinding

The study investigators and subjects were blinded with respect to drug/placebo status. The IDS provided this information to the principal investigator or medical personnel in the case of a patient’s medical emergency. If blinding had to be broken for this reason, the subject was to exit the study.

After completion of the full trial, individual participants were provided information from the IDS, indicating the order and dosing of treatment in their case. This allowed them and their parents the option of sharing their subjective experiences with their primary care provider, including any perceived benefits from LDX, in consideration of possibly pursuing further treatment on their own. However, the blinding of project staff with respect to treatment order was maintained for all cases throughout the study until completion of the final study subject.

#### 2.3.6. Note

Concomitant medications not listed in the exclusion criteria were permitted. No medications were changed or held for the purposes of entering the research study. If a child was started on a new medication by their medical provider, and that medication was on the list of excluded medications, the patient was to exit the study. Inquiry as to possible medication changes/additions were specifically assessed as part of the monitoring of safety and compliance in weekly visits with the study staff.

### 2.4. Neurobehavioral Assessments

All cases received a one-time assessment at baseline on the following measures. These were used as covariates or component measures facilitating interpretation on other tests.

- Abbreviated Wechsler Intelligence Scale for Children, Fifth Edition (WISC-V, general intelligence)
- Wisconsin Cart Sorting Test (WCST, set maintenance/shifting, executive functioning)
- Finger Oscillation (fine-motor speed/persistence)

The following are repeatable measures that were administered at baseline, 6 weeks (+/−3 days), and 12 weeks (+/−3 days):

- Conner’s Continuous Performance Test (CPT, sustained attention, delay, response modulation)
- Stroop Color-Word Test- Children’s Version (set maintenance/shifting, regulation of competing response tendencies)
- Letter & Animal Word Fluency, WISC-V Coding (processing speed/mental control)
- Woodcock-Johnson Understanding Directions (listening comprehension, following spoken instructions)
- WISC-V Digit Span (working memory)
- Wide Range Assessment of Memory and Learning-2 (WRAML): Verbal Learning and Design Memory subtests (short-term auditory-verbal memory and visual memory)
- Conners-3 Parent and Self-Report Forms (ratings of ADHD symptoms and related areas). Short-form versions of these were obtained during weekly visits.
- Child Behavior Checklist (CBCL, parent ratings of more general behavioral and emotional problems)
- Children’s Depression Inventory-2 (CDI, self-report of depression symptoms)
- Children’s Manifest Anxiety Scale-2 (RCMAS, self-report of anxiety symptoms)
- Behavior Rating Inventory of Executive Functioning-2 (BRIEF)—Parent and Self-Report Forms

#### Note

The above provided a broad-based assessment of cognition and behavioral/emotional status, as well as focusing on attention-related areas. It incorporated measures assessing the four components in Musky’s model of attention: focus-execute, sustain, encode, and shift [4]. Descriptions and normative data for many of the tests can be found in a *Compendium of Neuropsychological Tests* [17].

### 2.5. Data Analyses

The double blind, crossover design allowed for the assessment of both within-subjects and between-subjects contrasts. The primary analyses consisted of multiple paired-samples *t*-tests comparing LDX versus placebo on each of the neurobehavioral dependent measures. There was no power analysis based on sample size. Nor was there a formal correction applied for the multiple comparisons performed. As an initial study of its kind, the objective was to not limit sensitivity in detecting possible treatment effects. A *p*-value equal to, or less than, 0.05 was considered statistically significant. All tests were two-tailed. All analyses were performed using the Statistical Programs for the Social Sciences (IBM,2020,SPSS StatisticSubscription1.0.0.1327.Retrieved from https://www.ibm.com/products/spss-statistics/).

Possible order effects (depending on whether drug treatment came before or after placebo) were examined through a separate analysis of variance (ANOVA) for each dependent measure using a two-factor model (treatment, order, and treatment x order interaction).

There were also applications of analysis of covariance (ANCOVA) to determine possible mediating or moderating effects of pre-treatment variables on treatment outcomes (demographics, injury variables, IQ and other cognitive factors, behavioral symptoms, and other features).

Additionally, safety data were examined based on weekly visits over the course of the trial. Comparisons were made on LDX versus placebo for indices such as weight, blood pressure, and heart rate, side effects (insomnia, decreased appetite, etc.), or significant adverse events, if any. Each of these were examined using repeated measures ANOVA.

## 3. Results

### 3.1. Subject Characteristics

Table 1 provides a breakdown of the total sample of 20 cases in terms of selective demographic and clinical variables obtained at screening. The sample was evenly divided with respect to gender, but the racial composition consisted mainly of Caucasian children. The recruitment was open to children 6 years of age and older, but the actual sample ranged from 10 to 16 years of age. Parent reluctance to consider medication treatment for younger children was a factor in some cases.

Over half of the sample (55%) had sustained their head injuries in a sports-related activity. There were 25% whose injuries resulted from some type of MVA. The remainder of the sample was injured from other things, such as falls. There was an average duration of nearly 8 months since the time of injury, ranging from 2 months to 29 months at enrollment.

Noted in Table 1 are the mean scores pertaining to ADHD symptoms based on parent behavior ratings at screening. There were persistent post-concussion physical symptoms, especially headaches (50% of the cases) or fatigue/reduced stamina (35%). A quarter of the group had persistent physical symptoms in two or more areas (which also included such things as sleep disturbance, light sensitivity, etc.). There was a report of persistent emotional changes including increased irritability/frustration/temper problems (45%) or heightened anxiety/fearfulness/depression (25%). In addition, about one-third of the group were either having persistent struggles in school (drop in grades, increased effort, and/or stress over greater difficulty) or had the challenge of maintaining a record of high achievement or advanced studies. There was a premorbid history of concussion(s) in 40% of the sample, and 20% had some prior issues with attention (albeit less pronounced and without a diagnosis of ADHD).

This was entirely an outpatient subject sample at enrollment. Most had presented initially to the Vanderbilt Emergency Department/Pediatric Trauma Service shortly after their injury. They were diagnosed with a concussion/TBI either at that time or in a follow-up visit with a medical provider. The overall severity of TBI appeared to be mild in nearly all cases based on the available information (there was questionably a mild/moderate level of severity in 1–3 cases). A loss of consciousness (mostly brief) was noted in 30% of the sample (the rate was likely higher, but an adult observer often was not immediately present at the time of injury). There was a brief period of post-traumatic amnesia (estimated to be 30 min or less) in half of the group. It was more extended in about 10% of the cases. Glasgow Coma Scale scores, when available, fell in the 13–15 range (which, by convention, ordinarily corresponds to a mild level of TBI). Brain imaging had been performed on most of the cases, usually a head CT (computed tomography) scan on the day of the injury or shortly thereafter. Brain MRI (magnetic resonance imaging) scans were also done on several cases. Overall, the brain imaging findings were mostly read as normal. There were questionable acute findings in 2–4 of the cases. There were also two cases for whom there was a suggestion of an old malformation unrelated to acute injury. On the Post-TBI Symptom Questionnaire, significant cognitive problems were noted in three or more areas besides attention for 90% of the sample, as reported by either a parent or child. It was at 60% when reported by both the parent and child.

There were three cases who were fully enrolled but never began the trial, mostly due to scheduling problems. There was another case, an 11-year-old girl who withdrew due to a mild anxiety reaction (feeling “jumpy” and more easily overwhelmed) that began within one week after the crossover from placebo to LDX. She had taken the initial dose of 20 mg for a total of six days. Her symptoms subsided soon after she stopped the medication.

**Figure 1:**
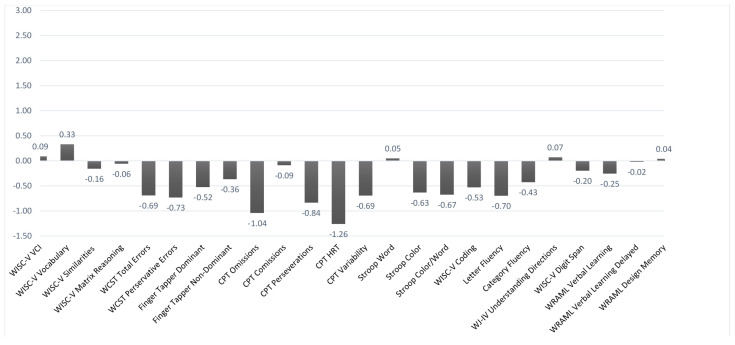
Performance Baseline Data. *n* = 17. The *y*-axis is based on *z*-scores (M = 0, SD = +/−1). Lower scores indicate poorer performance. Weschler Intelligence Scale for Children-5th ed. (WISC-V); Wisconsin Card Sorting Test (WCST); Conner’s Continuous Performance Test (CPT); Woodcock Johnson Test of Achievement-IV (WJ-IV); Wide Range Assessment of Memory and Learning (WRAML).

**Figure 2:**
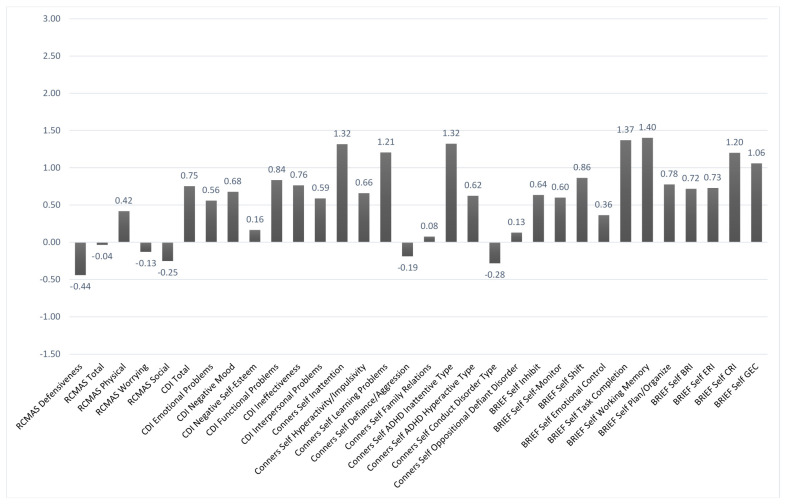
Behavioral Self-Report Baseline Data. *n* = 17. The *y*-axis is based on *z*-scores (M = 0, SD = +/−1). Higher scores indicate the reporting of greater problem. Revised Child Manifest Anxiety Scale (RCMAS); Child Depression Inventory (CDI); Behavior Rating Inventory of Executive Functioning (BRIEF); Behavior Regulation Index (BRI); Emotion Regulation Index (ERI); Cognitive Regulation Index (CRI); Global Executive Composite (GEC).

**Figure 3:**
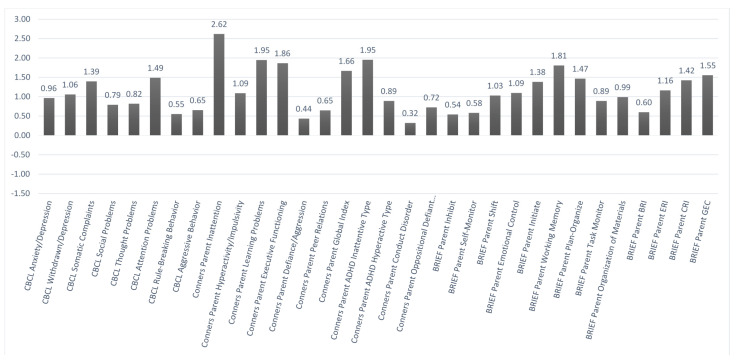
Behavioral Parent Report Baseline Data. *n* = 17. The *y*-axis is based on *z*-scores (M = 0, SD = +/−1). Higher scores indicate the reporting of greater problems. Child Behavior Checklist (CBCL); Behavior Rating Inventory of Executive Functioning (BRIEF); Behavior Regulation Index (BRI); Emotion Regulation Index (ERI); Cognitive Regulation Index (CRI); Global Executive Composite (GEC).

Figure 1, Figure 2 and Figure 3 illustrate the mean *z*-scores (where normative M = 0 and SD = +/−1) on the various cognitive and behavioral measures obtained at baseline (including the girl who later withdrew from the trial). Figure 1 deals specifically with the performance-based measures. For ease of interpretation, the layout was adjusted so that lower scores indicated poorer performance for all of the measures (higher scores on the Conner’s CPT ordinarily indicate poorer performance). Using a mean *z*-score cutoff of −1.5 or less, none of the performance measures fell significantly outside of a normal range based on the overall group results. There were slight to marginal trends in the case of mean CPT omissions and reaction time. However, when examined in terms of individual cases, there were varying rates of impairment across the different test measures. They were most prevalent with the CPT variables, especially omissions (for which 29.4% of the cases fell in an impaired range). About one-third of the baseline group (35.3%) had impaired scores on 2 or more of the subcomponents of the Conner’s CPT.

Figure 2 and Figure 3 show the mean *z*-scores on the various baseline behavioral measures based, respectively, on self-report and parent-report. In each case, higher scores indicated worse problems. Using a *z*-score cutoffof +1.5 or more, problems were noted in the areas of attentiveness, learning, and various aspects of executive functioning based mainly on the parent report. Similar problem areas were noted at baseline based on a self-report, but they fell more within a marginal range. That was seen in comparing the parent-report vs. self-report ratings on the Conners-3 Inattention scale (with *z*-scores of +2.62 vs. +1.32, respectively).

With respect to order of treatment, the randomization yielded an essentially even split of the subject group into the two conditions. That is, eight cases were assigned to the treatment order in which they received LDX in the first six weeks and placebo in the second six weeks, whereas nine cases received the opposite order. These subgroups were generally equivalent in terms of various screening variables noted in Table 1 and on the testing and behavior ratings performed at baseline.

### 3.2. Safety and Tolerability

As noted above, there was one case of a girl who withdrew from the trial due to an adverse event (AE) involving a report of increased anxiety. There was no other participant who met a safety endpoint due to either a medical or psychiatric AE.

For the remaining 16 subjects completing the trial, repeated measures ANOVAs on the weekly vitals data revealed a significant overall main effect for treatment (*p* = 0.023), with significant differences on/off LDX involving weight (*p* = 0.005), heart rate (*p* = 0.002), and diastolic blood pressure (DBP, *p* = 0.028). In addition, a significant treatment x time interaction was found for weight (*p* = 0.000), whereby, over time, participants gained weight when off treatment and lost weight when on treatment with LDX. Post hoc tests revealed that, during treatment with LDX, there was a significant weight loss by an average of 2.31 lbs. or 1.05 kg (*p* = 0.006), an increase in HR by an average of 7.46 beats per minute (*p* = 0.002), and an average increase in DBP of 3.37 mmHg (*p* = 0.028).

In terms of subjective reports of possible side-effects or tolerance issues during weekly visits, decreased appetite was the most common finding noted while on LDX (occurring in 75% of the cases). Irritability was the next most common complaint, but it was about equally prevalent whether on LDX or placebo (44% vs. 38%, respectively).

Of the 16 subjects who completed the trial, the average end dose on LDX was 53 mg whereas, on placebo, it was 69 mg. While on placebo, 15/16 cases went to the maximum dose of 70 mg and, except for one case who went for 1 week with a 10 mg rather than a 20-mg increase, they did that in the standard dose sequence. While on LDX, by contrast, there were only seven cases (44%) who progressed in the standard sequence to the maximum dose of 70 mg. For the remaining participants, there were tolerance issues that resulted in changes in the titration sequence such as stepping up in a 10 mg rather than a 20 mg increment or holding at a given dose for added week(s) or for the remainder of the LDX treatment. In some cases (31%), there was a roll-back to a previously tolerated dose. Three of the cases (19%) were maintained at the lower dose levels (20 mg–30 mg) for much, if not all, of the time on LDX.

### 3.3. Treatment Outcomes

Dependent-samples *t*-tests were performed comparing the results after 6 weeks of treatment with LDX vs. placebo on each of the neurobehavioral measures. Table 2 summarizes the main significant findings based on the 16 subjects completing the trial.

On LDX, there was a lower rate of omission errors on the Conner’s CPT, which is a measure of sustained attention or vigilance (*p* = 0.027). That was the only performance-based variable on which there was a significant treatment difference. There were positive treatment differences with LDX noted in terms of parent ratings of attention problems on the CBCL (*p* = 0.048) and on the Conners-3 dealing with parent ratings of hyperactivity/impulsivity (*p* = 0.024), ADHD inattentive symptoms (*p* = 0.037), and deficits in executive functioning (*p* = 0.034). In addition, on LDX, there was significantly better executive functioning as noted in parent behavior ratings spanning most of the areas assessed on the BRIEF-2.

Also noted in Table 2 are findings pertaining to the RCMAS, which is a self-report measure of anxiety. There were significantly higher *z*-scores on LDX vs. the placebo in terms of Total Symptoms (p = 0.012) and specifically on the Physical Symptoms subscale (p = 0.025). However, parent behavior ratings yielded a significantly lower score on the Anxious/Depressed scale of the CBCL while their child was on LDX vs. placebo (*p* = 0.027).

The order of treatment (whether LDX or placebo came first) made little difference in outcomes. No significant main effect for treatment order was found, but there were significant interactions for several dependent measures including Digit Span and Coding on the WISC-V (*p* = 0.026 and 0.039, respectively) and verbal learning on the WRAML (*p* = 0.004). In each case, performance was better with treatment mainly when it came in the second six weeks of the trial, possibly due to an enhancement of practice effects. In addition, there was a significant order effect with respect to self-report of Conner’s ADHD Inattentive symptoms such that greater improvement was reported when treatment with LDX treatment was in the second six weeks of the trial (*p* = 0.034). It may have been influenced by greater self-awareness as the child gained further experience in the trial.

### 3.4. Moderating Effects

Multiple ANCOVAs were performed with an extensive range of pre-treatment variables (demographics, clinical data, and test measures obtained at baseline) to determine possible predictive or moderating effects on treatment outcomes.

Significant moderating effects were found involving ratings of baseline inattentiveness based both on parent ratings on the Attention Problems scale of the CBCL (*p* = 0.01) and self-report ratings on the Conner’s scales (*p* = 0.01). In general, the presence of greater baseline CBCL Attention Problems scores was associated with better improvement on LDX vs. placebo in parent ratings of attentiveness and various aspects of executive functioning. Similarly, higher self-report ratings of inattentiveness at baseline were associated with better scores while on treatment with LDX in terms of parent ratings of different aspects of executive functioning on the BRIEF-2 as well as CBCL Anxious/Depressed ratings.

There was also a significant moderating effect involving baseline CBCL Anxious/Depressed scores (*p* = 0.052). Greater problems in this area noted by parents at baseline were associated with better improvement on LDX vs. placebo with such things as parent ratings of Inattentive Symptoms on the Conners Scales and different subscales of executive functioning on the BRIEF-2.

However, there were not robust or consistent moderating relationships found with respect to baseline performance measures including IQ. The same was true with respect to demographic variables including age and gender. In addition, there were not significant moderating effects on treatment outcomes having to do with the type of injury (sports, MVA, other), time since injury, or other factors noted at screening, such as whether there were persistent post-concussion physical symptoms, emotional disturbance, or school-related stress or whether there was a prior history of concussion(s) or attention issues.

## 4. Discussion

Positive treatment effects with LDX were found on various outcome measures, including performance-based testing of sustained attention. Thus, with respect to Mirsky’s model of different components of attention, it was the “sustain” element that was especially affected (there was a similar finding in the Tramontana et al. study with adults). In addition, there were positive treatment effects involving parent ratings of inattention, as well as hyperactive/impulsive behaviors, emotional status, and various aspects of executive functioning. Treatment differences based on self-report findings were less evident, perhaps because any pre-treatment problems noted at baseline tended to be less pronounced when reported by children versus their parents. Limited insight/self-awareness on the part of the child participants may have played a role in these differences based on the informant, which is not an unusual issue in child studies. However, for both children and their parents, the acknowledgement of greater attention problems at the outset predicted a better response to treatment with LDX on some of the outcome measures.

Treatment with LDX appeared to be generally safe when applied to this sample of children. LDX produced significant differences in weight, heart rate, and blood pressure, but not to an extent causing an adverse reaction. No participant met any of the pre-defined safety endpoints on vital signs during weekly monitoring. There was one participant who discontinued shortly after starting on treatment with LDX due to subjective complaints suggestive of mild anxiety. There were possible anxiety effects more generally within the sample, but the findings were inconsistent and of questionable significance. The higher scores obtained on the RCMAS, which is a self-report measure of anxiety, chiefly involved the Physical Symptoms subscale, which included items overlapping with some of the post-concussion physical symptoms within the group (headaches, nausea, fatigue, and sleep difficulty). The scores may not have been specifically reflective of anxiety, per se, but instead the sympathomimetic action of stimulant medication. In any event, behavior ratings by parents suggested that there were fewer problems with mood or anxiety while their child was on LDX versus the placebo. The most common side effect reported while on LDX was decreased appetite. Irritability was also relatively common, but it was reported about equally as often whether on LDX or placebo. Overall, there did not appear to be novel side-effects in this sample compared to stimulant effects ordinarily reported for children with idiopathic ADHD [18,19].

As far as the composition of the subject sample, there were rigorous selection criteria that resulted in the exclusion of many potential cases. Maintaining a trial of its length and complexity required a major time commitment by the participants. Recruitment was made challenging by other factors, such as resistance on the part of some parents to introducing medication as an intervention, which may have been an issue especially with younger children.

Factors such as the above likely limited or skewed the composition of the sample in some respects. Children below 10 years of age, although actively recruited, were not enrolled. Furthermore, there was a lack of diversity with respect to race even though recruitment was based in a large medical center serving diverse racial, ethnic, and socioeconomic groups. Possible reasons for that were unclear. Also noteworthy was that the sample consisted of children with TBIs of mainly mild severity. Cases with moderate to severe TBI were sought but their recruitment was limited mainly due to their involvement in other therapies or treatment interventions.

Despite the relatively mild severity of TBI in the subject group (at least as based on the known details of their injuries), there were persistent attention problems and other symptom lasting for as much as 29 months post-injury by the time of enrollment. Various factors may have been involved, including possible underestimation of TBI severity in some cases. Nearly all of the children had standard clinical brain imaging at or shortly after the time of injury (usually a head CT scan, which was typically read as negative). Several of the children had a brain MRI by the time of enrollment, which may have been more revealing with respect to severity and/or possible localization of injury if done more widely in the subject sample. This will be an issue to address more thoroughly in future studies, even in cases with seemingly milder injuries, especially when persisting symptoms are involved.

Interference from other factors may have played a contributing role. Conceivably, there may have been increased stress and fatigue over trouble focusing and keeping up, thereby causing an aggravation of post-concussion symptoms and, in turn, a persistence or worsening of attentions problems. In addition, there can be individual differences, such as sensitivity or reactivity to stress, that may intensify or accelerate such a chain of events.

In a recent study, Rieger et al. [20] compared adolescent students with and without concussion on a battery of neurocognitive, academic, and socio-emotional measures. The results indicated that the students reporting post-concussion symptoms did not perform differently than peers on most neurocognitive and academic measures, but they did show more worries, somatization, academic concerns, and feelings of inadequacy compared to matched controls. The findings served to highlight the importance of behavioral interventions to address psychological and academic stress that may be present in children and adolescents recovering from concussion. Moreover, other studies have identified factors involving parental behaviors and family functioning that can influence outcomes in children with TBI [21,22].

Problems with psychological and/or academic stress were reported for many of the children in the present study following their injuries. Persistent emotional problems of one kind or another were noted in 25% to 45% of the children at enrollment. About 35% were dealing with increased school-related stress. These, as well as other possible moderating factors affecting outcomes, were carefully examined, but the obtained findings were likely constrained by the limited size and variance within the subject sample. These will also be important issues to address in future studies.

## 5. Conclusions

There is a growing awareness of the impact of TBI on a child’s life, even when it involves milder injuries. There is a need for supportive assistance early on, including practical parent/child education concerning TBI as well as behavioral interventions where appropriate. Treatment with LDX appears to be a safe and effective option to consider. It can help to normalize attention processes and thereby modify a downward chain of events, such as the one noted above. The findings here suggest that it may benefit not only a child’s ability to sustain attention but, in doing so, can also impact positively on a broader range of functioning including behavioral and executive controls.

Overall, there are important avenues to build upon and further explore in going forward. It is hoped that the work done here will be extended to studies with a broader range of children with respect to demographics and more varied profiles of TBI. There is much to be learned both with respect to effective treatments as well as key factors to consider in monitoring the recovery process in a child with TBI.

## Figures and Tables

**Table 1:** Demographic and clinical information at enrollment.

Variable	Percentage	Mean (SD)	Range
Demographic:			
Gender	50% each		
Race	90% Caucasian		
Age		13.7 (2.39)	10–16
Grade level		8.2 (2.57)	4–12
Estimated IQ		104.5 (12.13)	85–120
Cause of injury:			
Sports	55%		
MVA	25%		
Other	20%		
Months since injury		7.85 (7.85)	2–29
Conners-3 Parent ADHD Inventory:			
Inattention		71.95 (12.93)	47–90
Hyperactivity/Impulsivity		65.10 (12.20)	42–90
Persistent Post Concussion Physical Symptoms:			
Headaches	50%		
Fatigue, reduced stamina	35%		
Persistent symptoms in 2 or more areas	25%		
Persistent Emotional Symptoms:			
Irritability/frustration/temper	45%		
Anxiety/fearfulness/depression	25%		
School Stress:			
Increased struggle/grade decline	35%		
High achievement record/advanced studies	35%		
Premorbid History:			
Concussion(s)	40%		
Attention issues	20%		

n = 20. Standard deviation (SD); Motor Vehicle Accident (MVA); Attention-Deficit/Hyperactivity Disorder (ADHD). Conner’s-3 Parent ADHD Inventory based on T-scores where M = 50, SD = 10; Estimated IQ based on standard scores where M = 100, SD = 15.

**Table 2:** Summary of the main treatment differences.

	Mean (SD)			
Variable	On Tx	Off Tx	*t*	*p-*Value
Conner’s CPT:				
Omission rate	0.625 (1.70)	1.35 (1.73)	−1.36	0.027
*RCMAS:*				
Physical symptoms	0.119 (0.796)	−0.219 (0.985)	2.49	0.025
Total symptoms	−0.3 (0.853)	−0.538 (0.920)	2.84	0.012
*CBCL:*				
Attention problems	0.881 (0.694)	1.33 (0.946)	−2.16	0.048
Anxiety/depression	0.444 (0.516)	0.788 (0.673)	−2.46	0.027
*BRIEF-2 Subscale-Parent:*				
Inhibit	−0.056 (1.05)	0.4 (1.20)	−2.28	0.038
Shift	0.325 (1.22)	0.925 (1.46)	−2.58	0.021
Initiate	0.644 (0.885)	1.18 (1.05)	−2.35	0.033
Working memory	0.963 (1.25)	1.66 (1.29)	−2.37	0.032
Task monitoring	0.338 (0.888)	0.744 (1.17)	−2.09	0.054
BRI	−0.44 (1.12)	0.444 (1.23)	−2.28	0.038
CRI	0.675 (1.02)	1.26 (1.32)	−2.20	0.044
ERI	0.35 (1.16)	0.888 (1.42)	−2.11	0.052
GEC	0.506 (0.993)	1.11 (1.35)	−2.28	0.038
*Conners-3 Subscale-Parent:*				
Hyperactivity/Impulsivity	0.075 (1.45)	0.569 (1.49)	−2.51	0.024
Executive functioning	0.969 (1.24)	1.67 (1.54)	−2.33	0.034
ADHD Inattentive Type	1.01 (1.12)	1.63 (1.40)	−2.30	0.037

*n* = 16. Treatment (Tx). Significant differences were based on *p* = 0.05 or less. The results are expressed as *z*-scores (M = 0, SD = +/−1), with higher scores indicating greater problems. CPT: Continuous Performance Test. RCMAS: Revised Child Manifest Anxiety Scale. CBCL: Child Behavior Checklist. BRIEF: Behavior Rating Inventory of Executive Functioning. BRI: Behavior Regulation Index. CRI: Cognitive Regulation Index. ERI: Emotion Regulation Index. GEC: Global Executive Composite.

## Data Availability

Data available on request due to restrictions. The data presented in this study are available on request from the corresponding author. The data are not publicly available due to privacy and ethical restrictions. Any release or sharing of data must preserve participant confidentiality and be approved by the Vanderbilt University Institutional Review Board for Humans Subjects Research.
